# What do Cochrane systematic reviews say about interventions for vitamin D supplementation?

**DOI:** 10.1590/1516-3180.2017.0230150817

**Published:** 2017-11-06

**Authors:** Mariana Vendramin Mateussi, Carolina de Oliveira Cruz Latorraca, Júlia Pozetti Daou, Ana Luiza Cabrera Martimbianco, Rachel Riera, Rafael Leite Pacheco, Daniela Vianna Pachito

**Affiliations:** I Undergraduate Medical Student, Escola Paulista de Medicina - Universidade Federal de São Paulo (EPM-Unifesp), São Paulo (SP), Brazil.; II MSc. Psychologist and Postgraduate Student, Postgraduate Evidence-Based Health Program, Universidade Federal de São Paulo (Unifesp), and Assistant Researcher, Cochrane Brazil, São Paulo (SP), Brazil.; III MSc, PhD. Physical Therapist and Assistant Researcher, Cochrane Brazil, São Paulo (SP), Brazil.; IV MD, MSc, PhD. Rheumatologist and Adjunct Professor, Discipline of Evidence-Based Medicine, Escola Paulista de Medicina - Universidade Federal de São Paulo (EPM-Unifesp); Assistant Coordinator, Cochrane Brazil, São Paulo (SP), Brazil.; V MD, MSc. Neurologist and Postgraduate Student, Postgraduate Evidence-Based Health Program, Universidade Federal de São Paulo (Unifesp), and Assistant Researcher, Cochrane Brazil, São Paulo (SP), Brazil.

**Keywords:** Review, Vitamin D, Evidence-based medicine, Evaluation of results of therapeutic interventions, Evidence-based practice

## Abstract

**CONTEXT AND OBJECTIVE:**

Despite the high prevalence of vitamin D supplementation, its use remains controversial. The objective of this review was to identify and summarize the evidence from Cochrane systematic reviews regarding vitamin D supplementation for preventing or treating any clinical condition.

**DESIGN AND SETTING:**

Review of systematic reviews, conducted in the Discipline of Evidence-Based Medicine, Escola Paulista de Medicina, Universidade Federal de São Paulo.

**METHODS:**

A search was conducted to identify all Cochrane systematic reviews that fulfilled the inclusion criteria. Titles and abstracts were screened by two authors.

**RESULTS:**

We included 27 Cochrane systematic reviews: 10 assessing use of vitamin D for prevention and 17 for treatment. The reviews found moderate to high quality of evidence regarding the benefit of vitamin D for pregnant women (prevention of adverse events: preterm birth risk [rate ratio, RR 0.36; 95% confidence interval, CI 0.14 to 0.93] and low birthweight risk [RR 0.40; 95% CI 0.24 to 0.67]) and for asthma patients (reduction of severe exacerbations [RR 0.63; 95% CI 0.45 to 0.88]). No benefit was found regarding vitamin D supplementation alone (without calcium) for preventing hip or any new fracture. For all other outcomes assessed under various conditions, the current quality of evidence is low or unknown, and therefore insufficient for any recommendation.

**CONCLUSION:**

Based on moderate to high quality of evidence, the Cochrane systematic reviews included here showed that there were some benefits from vitamin D supplementation for pregnant women and asthma patients and no benefits for preventing fractures.

## INTRODUCTION

A series of national surveys in the United States^1^ have shown that vitamin supplementation is done most commonly in relation to vitamin D. These analyses, relating to 2011-2012, showed that the prevalence of vitamin D supplementation was around 40%. After excluding supplementation using multivitamin or multimineral sources, the frequency of vitamin D supplementation was still high (approximately 19%).^2^ In addition, use of vitamin D supplementation has been increasing over the years. Supplementation with this micronutrient (excluding multivitamin/multimineral sources) was around 5.1% in 1999-2000 (with a difference of 14 percentage points from the 2011-2012 prevalence).^2^^,^^3^ The intake of high-dose vitamin D supplements (more than 1000 IU/day) has also increased. In 1999-2000, a prevalence of 0.3% was reported, versus 15.8% in 2011-2012.^4^

The number of published papers relating to vitamin D has also increased over time. A quick search in MEDLINE (via PubMed), using the strategy (“Vitamin D”[Mesh]) and a broad filter of clinical trials, retrieved 192 references in 2016, versus 75 in 1996 (a mean of 150 references per year was found over the period considered). Despite the popularity of vitamin D use and the high number of studies on this topic, the efficacy and safety of its supplementation are still a matter for debate in the literature.^5^ Because of the variety of dosages and formulations for vitamin D supplements and significant clinical heterogeneity between the conditions under which vitamin D is hypothesized to have benefits, the conclusions are still confusing.

Therefore, considering (a) the vast use of vitamin D supplementation, (b) the high number of primary studies published and (c) the controversy that continues in the literature, a systematic synthesis of the available evidence, such as the present review, is essential.

## OBJECTIVE

To identify and summarize the evidence from Cochrane systematic reviews regarding vitamin D supplementation for preventing or treating any clinical condition.

## METHODS

### Design and setting

A review of Cochrane systematic reviews was conducted within the Discipline of Evidence-based Medicine of Escola Paulista de Medicina, Universidade Federal de São Paulo (EPM-Unifesp). This article was specifically developed for the section Cochrane Highlights, which is an initiative for disseminating Cochrane reviews. This initiative results from a formal partnership between the São Paulo Medical Journal and Cochrane, and it is supported by Cochrane Brazil.

### Inclusion criteria

#### Types of study

We included any completed Cochrane systematic reviews published in the Cochrane Database of Systematic Reviews (CDSR). Protocols of systematic reviews and withdrawn or outdated versions of systematic reviews were not included. There was no limitation regarding date of publication.

#### Types of participants

Healthy individuals or those diagnosed with any clinical condition were included, regardless of age, ethnicity or sex.

#### Types of intervention

We considered systematic reviews on vitamin D supplementation, in any form (active or non-active), presentation (capsules or oral solution), dose (high, conventional or under-doses), regimen or duration of use, as a single or combined intervention. We did not consider reviews in which the clinical question involved vitamin D in combination with other interventions and in which it was not possible to assess the effect of vitamin D in isolation.

#### Types of outcomes

We considered any clinical or laboratory outcomes evaluated by the authors of the systematic reviews.

### Search for reviews

We conducted a systematic search in the Cochrane Database of Systematic Reviews (CDSR) (via Wiley) on April 4, 2017, sensitively using the MeSH term “Vitamin D” in titles, abstracts and keywords.

### Selection of reviews

The titles and abstracts were read by two out of three authors (MVM, RLP or DVP) independently. The systematic reviews that met the inclusion criteria was selected. Any disagreement was solved by consulting a further author (RR or COCL).

### Presentation of results

The results from the search and the systematic reviews included were presented through a descriptive structure (qualitative synthesis).

## RESULTS

### Search results

Our search strategy retrieved 53 references and, after screening the titles and abstracts, 28 systematic reviews were preselected. After assessing full texts, 27 reviews^6^^-^^32^ were found to fulfill our inclusion criteria and were included for qualitative analysis. One systematic review that assessed vitamin D plus calcium supplementation for treating corticosteroid-induced osteoporosis was excluded because it was not possible to evaluated the effect of vitamin D in isolation.^33^

### Reviews included

A summary of the issues, objectives, main findings and quality of evidence among the 27 systematic reviews included is presented below. We also present a brief individual summary of each review included, according to the clinical situation. For detailed explanations, the full versions can be viewed in the box in Table 1.^6^^-^^32^^,^^34^

**Table 1.**
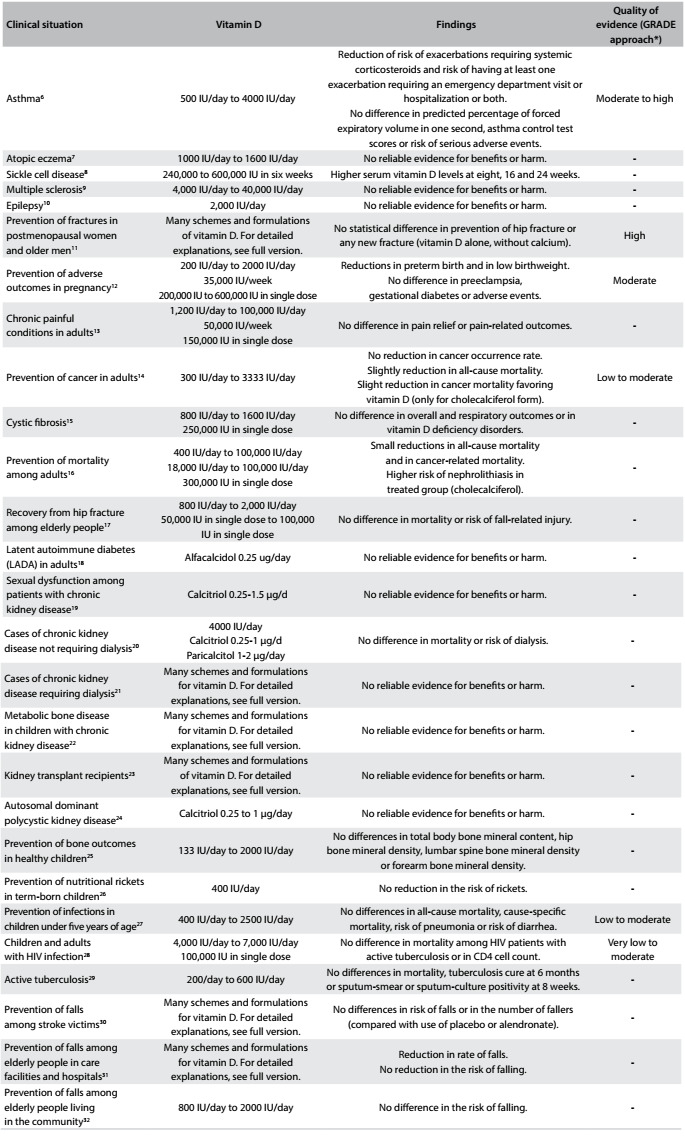
Main characteristics relating to clinical situation, intervention, findings and quality of evidence among the systematic reviews included

*GRADE^34^ (Grading of Recommendations Assessment, Development and Evaluation) is used for assessment of the quality of the body of evidence.

#### 1. *Asthma*

The review^6^ evaluated the effect of vitamin D for preventing asthma attacks or improving disease control, in comparison with placebo or no intervention. Nine randomized clinical trials (RCTs) involving 1,093 participants were included. The majority of the participants were considered to have mild to moderate asthma. The results showed that vitamin D presents benefits relating to:

- Risk of exacerbations requiring systemic corticosteroids (number of events per participant per year, during a follow-up of six to 12 months (rate ratio, RR 0.63; 95% confidence interval, CI 0.45 to 0.88; three RCTs; 680 participants; high-quality evidence);
- Risk of experiencing at least one exacerbation requiring an emergency department visit or hospitalization or both (odds ratio, OR 0.39; 95% CI 0.19 to 0.78; seven RCTs; 963 participants; high-quality evidence).

There was no difference between the intervention groups for the following outcomes: predicted percentage of forced expiratory volume in one second (mean difference, MD 0.48; 95% CI -0.93 to 1.89; four RCTs; 387 participants; high-quality evidence); asthma control test scores (MD -0.08; 95% CI -0.70 to 0.54; three RCTs; 713 participants; high-quality evidence); and risk of serious adverse events (OR 1.01; 95% CI 0.54 to 1.89; five RCTs, 879 participants; moderate-quality evidence). These results should be taken carefully and their clinical relevance needs to be assessed by an asthma specialist, preferably supported by cost-effectiveness analysis, before any recommendation for practice is made.

For further details, refer to the original abstract, available at: http://onlinelibrary.wiley.com/doi/10.1002/14651858.CD011511.pub2/full.

#### 2. *Atopic eczema*

The review^7^ evaluated dietary supplementation, including vitamin D, for treatment of atopic dermatitis. Among the 11 RCTs included in the full review, two studies (n = 63) evaluated the effects of vitamin D supplementation, and showed that there was no benefit from vitamin D use for primary efficacy outcomes (participant/parent-rated symptoms of atopic eczema, such as pruritus or sleep loss, or reduction in the number of flares or need for other treatments) or secondary efficacy outcomes (overall severity assessed by participants or physicians, quality of life or adverse events). Both studies had limited numbers of participants, overall low methodological quality and short follow-up periods, which precluded any definitive conclusion regarding the effect of vitamin D on atopic dermatitis. Therefore, the authors concluded that the current evidence was insufficient for routinely recommending vitamin D for such clinical situations in practice.

For further details, refer to the original abstract, available at: http://onlinelibrary.wiley.com/doi/10.1002/14651858.CD005205.pub3/full.

#### 3. *Sickle cell disease*

Sickle cell disease is associated with multiple micronutrient deficiencies. The review^8^ aimed to investigate the effects of vitamin D supplementation on sickle cell disease and only one RCT (39 participants) was found.

Compared with placebo, the vitamin D group had significantly higher serum vitamin D levels after:

- Eight weeks (MD 29.79; 95% CI 26.63 to 32.95; 37 participants; moderate-quality evidence);
- 16 weeks (MD 12.67; 95% CI 10.43 to 14.90);
- 24 weeks (MD 15.52; 95% CI 13.50 to 17.54).

No statistical significance was found in relation to any other outcome evaluated through this RCT. This study was classified as containing a high risk of attrition bias, because only 25 of the 37 randomized participants (67%) completed the six-month follow-up. The authors concluded that no practical recommendation could be made regarding supplementation of vitamin D among patients with sickle cell disease.

For further details, refer to the original abstract, available at: http://onlinelibrary.wiley.com/doi/10.1002/14651858.CD010858.pub2/full.

#### 4. *Multiple sclerosis*

The review^9^ evaluated the effects of vitamin D for multiple sclerosis and included only one small RCT (n = 49; 52-week follow-up), which compared serial doses of vitamin D versus placebo. The authors reported that there was a reduction in the relapse rate, a higher proportion of relapse-free patients and a reduction in the EDSS score (i.e. the disability score) in the intervention group. However, no numerical data were provided. In additional, the RCT included had a small sample size and significant methodological limitation, and so presented substantial risk of bias. Therefore, the current evidence did not provide any solid conclusion regarding the effects of vitamin D on multiple sclerosis.

For further details, refer to the original abstract, available at: http://onlinelibrary.wiley.com/doi/10.1002/14651858.CD008422.pub2/full.

#### 5. *Epilepsy*

The review^10^ assessed the effects of different interventions on epilepsy and included 15 RCTs, but only two of them (n = 274) evaluated the use of vitamin D. However, no data were available regarding freedom from seizures, seizure frequency, quality of life, cognitive function, reduced side effects from antiepileptic drugs or any other outcome considered by the review. The studies had significant methodological limitation and therefore high risk of bias. The authors’ conclusion was that no reliable evidence was found to support use of vitamin D among epileptic patients.

For further details, refer to the original abstract, available at: http://onlinelibrary.wiley.com/doi/10.1002/14651858.CD004304.pub2/full.

#### 6. *Postmenopausal women and older men*

The purpose of the review^11^ was to determine the effects of vitamin D or related compounds for preventing fractures among postmenopausal women and older men. This review included 53 RCTs (n = 91,791) and the results showed that vitamin D alone was not effective for preventing:

- Hip fracture (RR 1.12; 95% CI 0.98 to 1.29; 11 RCTs; n = 27,693);
- Any new fracture (RR 1.03; 95% CI 0.96 to 1.11; 15 RCTs; n = 28,271).

The authors concluded that with the current evidence, vitamin D alone was unlikely to prevent fractures.

For further details, refer to the original abstract, available at: http://onlinelibrary.wiley.com/doi/10.1002/14651858.CD000227.pub4/full.

#### 7. *Prevention of adverse outcomes in pregnancy*

The aim of the systematic review^12^ was to assess the effects of oral supplementation with vitamin D during pregnancy for improving maternal and neonatal outcomes. The review included 15 RCTs (n = 2,833) and it showed that vitamin D supplementation during pregnancy provided benefits regarding the following outcomes:

- Risk of preterm birth (RR 0.36; 95% CI 0.14 to 0.93; three RCTs; n = 477; moderate-quality evidence);
- Risk of low birthweight (RR 0.40; 95% CI 0.24 to 0.67; three RCTs; n = 493; moderate-quality evidence).

There was no statistical difference between the groups regarding the risk of preeclampsia (RR 0.52; 95% CI 0.25 to 1.05; two RCTs; n = 219; low-quality evidence), gestational diabetes (RR 0.43; 95% CI 0.05 to 3.45; two RCTS; n = 219; very low-quality evidence) or adverse effects (RR 0.17; 95% CI 0.01 to 4.06; one RCT; n = 135; low-quality evidence). In the light of these results, the authors concluded that the benefits of vitamin D supplementation as part as routine antenatal care were still unclear and that further studies were needed in order to confirm whether there was any benefit in relation to maternal and neonatal outcomes.

For further details, refer to the original abstract, available at: http://onlinelibrary.wiley.com/doi/10.1002/14651858.CD008873.pub3/full.

#### 8. *Chronic painful conditions in adults*

The review^13^ assessed the efficacy and safety of vitamin D supplementation versus placebo or versus active comparators, for adults with chronic painful conditions. Ten RCTs (n = 811), including people with rheumatoid arthritis (four RCTs), knee osteoarthritis (two RCTs), polymyalgia rheumatic (one RCT), “non-specific” musculoskeletal pain (one RCT), “diffuse” musculoskeletal pain (one RCT) and fibromyalgia (one RCT), were included. These studies were very heterogeneous regarding clinical and methodological characteristics, and therefore no quantitative analysis was possible. No statistical difference was demonstrated for any primary outcome (number of participants with 50% pain relief, improvement in overall impression of pain change or any other pain-related outcome). Based on these results, no recommendation for any vitamin D supplementation for chronic painful conditions could be made.

For further details, refer to the original abstract, available at: http://onlinelibrary.wiley.com/doi/10.1002/14651858.CD007771.pub3/full.

#### 9. *Prevention of cancer in adults*

The aim of the review^14^ was to assess the effectiveness and safety of vitamin D (cholecalciferol, ergocalciferol, alfacalcidol and calcitriol) for preventing cancer, and included 18 RCTs (n = 50,623). The following results were found:

- Risk of cancer: no significant reduction through vitamin D supplementation over a follow-up of 0.5 to seven years (RR 1.00; 95% CI 0.94 to 1.06; 18 RCTs; n = 50,623; moderate-quality evidence);
- All-cause mortality: through vitamin D supplementation over a follow-up of 0.5 to 7 years (RR 0.93; 95% CI 0.88 to 0.98; 15 RCTs; n = 49,866; low-quality evidence);
- Cancer-related mortality: no significant reduction through cholecalciferol (RR 0.88; 95% CI 0.78 to 0.98; four RCTs; n = 44,492; low-quality evidence).

This review did not show any evidence of effects from any presentation of vitamin D in relation to reducing the risk of cancer. It was found that there was a general need for more studies in order to make any recommendations regarding clinical practice.

For further details, refer to the original abstract, available at: http://onlinelibrary.wiley.com/doi/10.1002/14651858.CD007469.pub2/full.

#### 10. *Cystic fibrosis*

The review^15^ examined the effects of vitamin D supplementation for cystic fibrosis patients, and included six randomized and quasi-randomized controlled studies (n = 239). No improvements in general or respiratory outcomes or in vitamin D deficiency disorders (osteopenia or osteoporosis) were found. The high clinical and methodological heterogeneity between studies precluded meta-analysis. The number of published studies was small and there was currently no evidence of clinical benefit from vitamin D supplementation.

For further details, refer to the original abstract, available at: http://onlinelibrary.wiley.com/doi/10.1002/14651858.CD007298.pub4/full.

#### 11. *Prevention of mortality among adults*

The objective of the review^16^ was to assess the benefits and harm from vitamin D supplementation in relation to preventing mortality. It included 159 RCTs, but only 56 (n = 95,286) reported usable data regarding mortality. The following results were found for the comparisons of cholecalciferol versus placebo or no intervention:

- All-cause mortality: reduction of 6% over a seven-year follow-up [(RR 0.94; 95% CI 0.91 to 0.98; number needed to treat (NNT) = 150; 38 RCTs; n = 75,927; moderate-quality evidence)];
- Cancer-related mortality; reduction of 12% (RR 0.88; 95% CI 0.79 to 0.98; four RCTs; n = 44,492; moderate-quality evidence).

For the comparisons of cholecalciferol plus calcium versus placebo or no treatment, the risk of nephrolithiasis was higher in the treated group (RR 1.07; 95% CI 1.02 to 1.34; four RCTs; n = 42,876). The risk of attrition bias due to the substantial dropout of participants and the risk of outcome reporting bias regarding mortality weakened the current evidence. Further RCTs with strong methodological and reporting quality would be needed in order to draw any practical conclusion.

For further details, refer to the original abstract, available at: http://onlinelibrary.wiley.com/doi/10.1002/14651858.CD007470.pub3/full.

#### 12. *Recovery from hip fracture among elderly people*

The aim of the review^17^ was to evaluate the effects of nutritional interventions among elderly people who were recovering from hip fractures. It included 24 RCTs, but only four RCTs evaluated vitamin D supplementation. There was no evidence of any effects from vitamin D supplementation regarding any primary or clinical outcomes such as mortality or the risk of fall-related injury. Currently, there was no evidence that vitamin D had any beneficial effect among older patients who were recovering from hip fractures.

For further details, refer to the original abstract, available at: http://onlinelibrary.wiley.com/doi/10.1002/14651858.CD001880.pub6/full.

#### 13. *Latent autoimmune diabetes (LADA) in adults*

The review^18^ aimed to assess the effects of different interventions relating to latent autoimmune diabetes (LADA), which is a kind of type 1 diabetes with slow progression to insulin dependency. Ten RCTs (n = 1,109) were included, but only one related to vitamin D. This study compared vitamin D plus insulin versus insulin alone and showed that the peptide C levels in the combined group remained steady, but that these levels decreased in the insulin-alone group (368 to 179 pmol/L; P = 0.006) over 12 months of follow-up. The lack of methodologically high-quality studies prevented any practical recommendation regarding the use of vitamin D for treatment of LADA.

For further details, refer to the original abstract, available at: http://onlinelibrary.wiley.com/doi/10.1002/14651858.CD006165.pub3/full.

#### 14. *Sexual dysfunction among patients with chronic kidney disease (CKD)*

The purpose of the review^19^ was to evaluate the benefits and harm of interventions for treating sexual dysfunction among CKD patients. It included 15 RCTs (n = 352 patients), but only one RCT (n = 15) aimed to assess the efficacy of vitamin D. No sound benefits were found among the outcomes reported (endocrine parameter levels) in this study. The small sample size and the high risk of bias attributed to this study prevented any conclusion regarding the use of vitamin D for treating sexual dysfunction among CKD patients.

For further details, refer to the original abstract, available at: http://onlinelibrary.wiley.com/doi/10.1002/14651858.CD007747.pub2/full.

#### 15. *Cases of chronic kidney disease (CKD) not requiring dialysis*

The objective of the review^20^ was to evaluate the efficacy of vitamin D for patients with CKD who were not requiring dialysis. Sixteen RCTs (n = 894) were included. Some analyses showed lower serum parathyroid hormone (PTH) concentration and higher calcium and phosphorus concentration, but no benefits in relation to primary clinical outcomes, such as mortality or risk of dialysis, were found. The authors concluded that there were insufficient data to determine the effect of vitamin D on mortality and relevant clinical outcomes among chronic non-dialytic renal patients.

For further details, refer to the original abstract, available at: http://onlinelibrary.wiley.com/doi/10.1002/14651858.CD008175/full.

#### 16. *Cases of chronic kidney disease (CKD) requiring dialysis*

The review^21^ aimed to evaluate the efficacy and safety of vitamin D compounds in CKD patients requiring dialysis. It included 60 RCTs (n = 2,773) that evaluated multiple formulations, routes and schedules for administration of vitamin D compounds. No clinically relevant outcomes favored the intervention group in any comparisons. Some analyses showed decreased serum PTH levels and a tendency towards higher calcium and phosphate concentrations. In the light of the low quality and poor reporting, and considering the marked clinical and methodological heterogeneity of the studies available, no strong practical recommendations could be made.

For further details, refer to the original abstract, available at: http://onlinelibrary.wiley.com/doi/10.1002/14651858.CD005633.pub2/full.

#### 17. *Metabolic bone disease in children with chronic kidney disease (CKD)*

The purpose of the review^22^ was to examine the benefits and harm of interventions for prevention and treatment of metabolic bone disease in children with CKD. This review included 18 RCTs (n = 576) and evaluated eight different interventions, most of them including at least one vitamin D preparation. Other than differences in PTH levels between groups in some of these comparisons, no other clinical or primary outcome was improved through vitamin D supplementation. Therefore, no solid recommendation in favor of vitamin D supplementation could be made.

For further details and more information about the comparisons included, refer to the original abstract, available at: http://onlinelibrary.wiley.com/doi/10.1002/14651858.CD008327.pub2/full.

#### 18. *Kidney transplant recipients*

The aim of the review^23^ was to evaluate the use of interventions for preventing bone disease following kidney transplantation. Twenty-four RCTs (n = 1,299) were included. Studies that assessed use of vitamin D sterols and other interventions such as use of bisphosphonates, calcitonin and other substances were considered. The analysis on vitamin D sterol alone versus placebo or no treatment did not show any reduction in the risk of fractures or other clinical outcomes such as all-cause mortality. Some improvement in bone mineral density (lumbar spine and femoral neck) was reported in one RCT. Because of the lack of studies of high methodological quality assessing the use of vitamin D alone for preventing bone disease, it was difficult to come to any practical conclusion before further studies investigating this question are developed.

For further details, refer to the original abstract, available at: http://onlinelibrary.wiley.com/doi/10.1002/14651858.CD005015.pub3/full.

#### 19. *Autosomal dominant polycystic kidney disease (ADPKD)*

The review^24^ evaluated the effects of interventions for preventing progression of ADPKD, in relation to kidney function, kidney endpoints, kidney structure, patient-centered endpoints and the adverse effects of these treatments. This review included 30 studies, but only one RCT (n = 34) evaluated vitamin D supplementation, which was done in comparison with Chinese medicine (herbs). The data were sparse and inconclusive, and the authors were unable to make any solid recommendations.

For further details, refer to the original abstract, available at: http://onlinelibrary.wiley.com/doi/10.1002/14651858.CD010294.pub2/full.

#### 20. *Prevention of bone outcomes in healthy children*

The aim of the review^25^ was to determine the effectiveness of vitamin D supplementation for increasing bone mineral density in children. Six RCTs were included (n = 884). No statistically significant differences between the vitamin D and control groups were found in relation to:

- Total body bone mineral content (standard mean difference, SMD 0.1; 95% CI -0.06 to 0.26; 5 RCTs; n = 672; high-quality evidence);
- Hip bone mineral density (SMD 0.06; 95% CI -0.18 to 0.29; 4 RCTs; n = 639; moderate-quality evidence);
- Lumbar spine bone mineral density (SMD 0.15; 95% CI -0.01 to 0.31; 5 RCTs; n = 660);
- Forearm bone mineral density (SMD 0.04; 95% CI -0.36 to 0.45; 3 RCTs; n = 427).

The authors concluded that the results did not support supplementation to improve bone mineral in children.

For further details, refer to the original abstract, available at: http://onlinelibrary.wiley.com/doi/10.1002/14651858.CD006944.pub2/full.

#### 21. *Prevention of nutritional rickets in term-born children*

The review^26^ aimed to evaluate the effects of any intervention for prevention of rickets in term-born children. It included three RCTs and one non-randomized clinical trials (n = 1,700). Only three studies evaluated use of vitamin D alone, but in two of these studies there were no occurrences of rickets in either group. One study comparing vitamin D (400 IU per day for 12 months) versus no intervention showed that there was a statistical reduction in the risk of occurrence of rickets (RR 0.04; 95% CI 0 to 0.71; one RCT; n = 676). Even in the light of this result, the authors considered it reasonable to offer vitamin D or calcium for prevention of rickets to all children up to two years of age. However, they pointed out that further studies would be needed to investigate the effects in higher-risk subgroups and to investigate possible adverse effects.

This recommendation needs to be better evaluated, considering that

1. Only one study contributed towards this conclusion;
2. The evaluation on the risk of bias for this systematic review, published in 2007, did not follow the current standard (i.e. the risk-of-bias table from the Cochrane Collaboration); and
3. No assessment of the quality of the body of the evidence was required by the Cochrane Collaboration at the time when this systematic review was published.

For further details, refer to the original abstract, available at: http://onlinelibrary.wiley.com/doi/10.1002/14651858.CD006164.pub2/full.

#### 22. *Prevention of infections in children under five years of age*

The purpose of the review^27^ was to evaluate the role of vitamin D supplementation in preventing infections in children under five years of age. Four RCTs (n = 3,198) were included. No difference between the vitamin D and control groups (placebo or no supplementation) was found in relation to the following outcomes:

- All-cause mortality (mortality due to any cause of death) (RR 1.43; 95% CI 0.54 to 3.74; one RCT; n = 3,046; low-quality evidence);
- Cause-specific mortality (mortality due to pneumonia, tuberculosis, diarrhea or malaria) (RR 1.50; 95% CI 0.42 to 5.30; one RCT; n = 3,046; low-quality evidence);
- Risk of a radiologically confirmed first or only episode of pneumonia (RR 1.06; 95% CI 0.89 to 1.26; two RCTs; n = 3,134; moderate-quality evidence);
- Risk of diarrhea (two RCTs; no numerical data provided).

No study investigating other infectious diseases such as tuberculosis or malaria was found. The authors concluded that the implications of this systematic review were limited because of the low availability of primary studies.

For further details, refer to the original abstract, available from: http://onlinelibrary.wiley.com/doi/10.1002/14651858.CD008824.pub2/full.

#### 23. *Children and adults with HIV infection*

The review^28^ aimed to evaluate the efficacy and safety of micronutrient supplementation for reduction of morbidity and mortality among adults and children with HIV infection. Thirty-three RCTs were included, but only five (n = 447) were on vitamin D (alone or in combination with calcium). The results showed that vitamin D did not provide any benefit in relation to the following outcomes:

- Mortality among HIV patients with active tuberculosis (RR 1.15; 95% CI 0.65 to 2.02; one RCT; n = 131; very low-quality evidence);
- CD4 cell count (data from four RCTs, not pooled; n = 288).

The authors concluded that further larger RCTs, with strong methodological quality, using individual supplementation of vitamin D would be needed in order to build a baseline of evidence.

For further details, refer to the original abstract, available at: http://onlinelibrary.wiley.com/doi/10.1002/14651858.CD003650.pub4/full.

#### 24. *Active tuberculosis*

The objective of the review^29^ was to assess the effects of oral nutritional supplements among individuals receiving anti-tuberculosis drug therapy to treat active tuberculosis. Eleven RCTs evaluated the effects of vitamin D supplementation. The results showed that vitamin D did not provide any benefit in relation to the following:

- Mortality (RR 0.96; 95% CI 0.81 to 1.12; seven RCTs; n = 2,649);
- Tuberculosis cure at six months (RR 0.99; 95% CI 0.75 to 1.31; one RCT; n = 151);
- Sputum-smear or sputum-culture positivity at eight weeks (RR 0.81; 95% CI 0.54 to 1.20; six RCTs; n = 856).

The authors concluded that there was no reliable evidence that would support vitamin D supplementation among people who were being treated for active tuberculosis.

For further details, refer to the original abstract, available at: http://onlinelibrary.wiley.com/doi/10.1002/14651858.CD006086.pub4/full.

#### 25. *Prevention of falls among stroke victims*

Falls are a common complication after stroke. Around 7% of stroke victims suffer falls in the first week after stroke onset. The objective of the review^30^ was to evaluate the effectiveness of interventions for preventing falls among these individuals. It included ten RCTs, but only two were on vitamin D. One RCT (n = 85) compared vitamin D versus placebo among women who had been institutionalized after suffering a stroke, and now presented low serum vitamin D levels. The second RCT compared alfacalcidol versus alendronate among individuals who were hospitalized after stroke. For both studies, there was no statistical difference between the vitamin D and comparison groups regarding the risk of falls and the number of fallers. The authors concluded that these data should be considered provisional until further studies had been developed.

For further details, refer to the original abstract, available at: http://onlinelibrary.wiley.com/doi/10.1002/14651858.CD008728.pub2/full.

#### 26. *Prevention of falls among elderly people in care facilities and hospitals*

The review^31^ aimed to evaluate the effectiveness of interventions to reduce falls among elderly people. It included 60 RCTs (43 conducted in healthcare facilities and 17 in hospitals), totaling 60,345 participants. In the healthcare facilities, the vitamin D group had a lower fall rate, defined as the number of falls per person-year (RR 0.63; 95% CI 0.46 to 0.86; five RCTs; n = 4,603), but did not have a lower risk of falling, defined as the number of people falling (i.e. fallers) (RR 0.99; 95% CI 0.9 to 1.08; six RCTs; n = 5,186). No measurement of the quality of the body of evidence was reported in this systematic review. The authors concluded that, in care facilities, vitamin D supplementation seemed to be effective in reducing the fall rate.

For further details, refer to the original abstract, available at: http://onlinelibrary.wiley.com/doi/10.1002/14651858.CD005465.pub3/full.

#### 27. *Prevention of falls among elderly people living in the community*

The review^32^ aimed to evaluate the effects of interventions to prevent falls among the elderly population. The authors included 159 RCTs, but only 7 were on vitamin D (n = 9,324). No benefit from vitamin D regarding the risk of falls was found (RR 0.96; 95% CI 0.89 to 1.03; 13 RCTs; n = 26,747). The authors concluded that vitamin D supplementation did not seem to reduce the number of falls.

For further details, refer to the original abstract, available at: http://onlinelibrary.wiley.com/doi/10.1002/14651858.CD007146.pub3/full.

## DISCUSSION

This study included 27 Cochrane systematic reviews: 10 related to prevention and 17 assessed use of vitamin D as a therapeutic option. Among the preventive systematic reviews, five focused on healthy individuals for prevention of death,^16^ cancer,^14^ and other adverse outcomes.^12^^,^^25^^,^^27^ Despite the considerable number of Cochrane reviews, eight of them did not present any reliable evidence regarding benefits or harm from vitamin D, including reviews on clinical situations in which use of vitamin D is frequently observed in clinical practice, such as in relation to multiple sclerosis^9^ and atopic eczema.^7^ Thus, in those reviews, the authors were unable to provide recommendations regarding the benefits and risks of vitamin D.

Currently, there are major concerns and inconsistencies regarding vitamin D use in clinical practice. Although its use has been disseminated by the media, and is widespread among healthcare professionals and patients, there are few clinical studies supporting this practice. Moreover, the existing studies present poor methodological quality, thus leading to uncertain results.

It is important to highlight that most of the trials providing the current evidence were methodologically inappropriate due to small sample sizes, insufficient follow-up periods or unclear or inadequate methods for randomization and blinding. Furthermore, there was huge clinical and methodological heterogeneity among clinical trials with the same PICO (P = population, I = intervention, C = comparison, O = outcomes), which precluded additional quantitative synthesis. All of these features limited the quality of current available evidence for relevant outcomes.

A second point that deserves discussion is that many of the reviews included were planned, conducted or published some years ago, and therefore were based on early versions of the Cochrane Handbook, which were less detailed and complex than the current version (http://training.cochrane.org/handbook). As an example, the inclusion criteria and methods for assessing the quality of clinical trials were not refined as they are now. Moreover, Grading of Recommendations Assessment, Development and Evaluation (GRADE) is a new approach that was not yet mandatory for Cochrane reviews. Implementation of the Cochrane Risk of Bias Table, and mandatory use of GRADE and Methodological Expectations of Cochrane Intervention Reviews (MECIR) have contributed towards improving the methodological rigor of Cochrane reviews over the years. Therefore, it is reasonable to assume that older reviews may not present the same quality as recent reviews, which could limit the applicability of findings.

Regarding the implications of these Cochrane reviews for clinical practice, moderate to high-quality evidence of benefit from vitamin D for pregnant women (prevention of maternal and child adverse outcomes) and for asthma patients (reduction of severe exacerbations) was found. Additionally, no benefit from vitamin D supplementation alone (without calcium) for preventing hip fracture or any new fracture was found. For all other benefits or harm, the current quality of evidence was low or unknown, and therefore insufficient for any recommendations.

Regarding the implications of this study for further research, it was found that well-designed and well-conducted randomized clinical trials are essential for assessing the effectiveness and safety of vitamin D for many clinical situations, in which ‘off-label use’ is already commonly observed in clinical practice. Moreover, a number of core questions need to be addressed, including determining definitions for the following: the normal serum levels of vitamin D; the most appropriate type of vitamin D supplementation; the optimal doses for vitamin D supplementation; the most accurate method for assessing serum vitamin D levels; and the relationship between vitamin D and the risk of diseases.

## CONCLUSION

This study identified 27 Cochrane reviews that provided evidence of quality ranging from unknown to high, in relation to vitamin D supplementation as preventive or therapeutic intervention. Vitamin D was found to present some benefit for pregnant women and asthma patients and no benefit when administered alone (without calcium) for preventing fractures. For all other likely benefit or harm that has been evaluated through Cochrane reviews, the current quality of evidence is low or unknown, and therefore insufficient for any sound conclusion.
